# LncRNA TNFRSF10A-AS1 promotes gastric cancer by directly binding to oncogenic MPZL1 and is associated with patient outcome

**DOI:** 10.7150/ijbs.68776

**Published:** 2022-05-01

**Authors:** Donglei Sun, Hongyan Gou, Dandan Wang, Chenyang Li, Yan Li, Hao Su, Xiaohong Wang, Xiaolan Zhang, Jun Yu

**Affiliations:** 1 Department of Gastroenterology, The Second Hospital of Hebei Medical University, No. 215 Heping West Road, Shijiazhuang 050000, Hebei, China.; 2 Shenzhen Research Institute, The Chinese University of Hong Kong, Shenzhen, China.; 3 Institute of Digestive Disease and Department of Medicine and Therapeutics, State Key laboratory of Digestive Disease, Li Ka Shing Institute of Health Sciences, The Chinese University of Hong Kong, Hong Kong SAR, China.; 4 Key laboratory of Carcinogenesis and Translational Research, Peking University Cancer Hospital and Institute, Beijing, China.

**Keywords:** TNFRSF10A-AS1, Long non-coding RNA, Gastric cancer, MPZL1

## Abstract

**Background:** LncRNA is closely associated with the progression of human tumors. The role of lncRNA TNFRSF10A-AS1 (T-AS1) in gastric cancer (GC) is still unclear. We aim to investigate the functional significance and the underlying mechanisms of T-AS1 in the pathogenesis and progression of GC.

**Experimental Design:** The clinical impact of T-AS1 was assessed in 103 patients with GC. The biological function of T-AS1 was studied *in vitro* and *in vivo*. T-AS1 downstream effector were identified by RNA sequencing and RNA pulldown assay.

**Results:** T-AS1 was upregulated in GC cell lines and GC tissues as compared to adjacent non-cancer tissues (n = 47, *P* < 0.001). Multivariate analysis showed that GC patients with T-AS1 high expression had a significantly shortened survival (n=103,* P* < 0.05). T-AS1 significantly promoted GC cell proliferation, cell-cycle progression, and cell migration/invasion abilities, but suppressed cell apoptosis. Silencing of T-AS1 in GC cells exerted opposite effects *in vitro*. Knockout of T-AS1 significantly inhibited xenograft tumor growth in nude mice. Mechanistically, T-AS1 directly bound to Myelin Protein Zero Like 1 (MPZL1). MPZL1 showed an oncogenic function in GC by promoting cell proliferation, migration and invasion but inhibiting cell apoptosis. High expression of MPZL1 was associated with poor survivor of GC patients. Knockdown of MPZL1 could abrogate the effect of T-AS1 in the tumor-promoting function.

**Conclusions:** T-AS1 plays a pivotal oncogenic role in GC and is an independent prognostic factor for GC patients. The oncogenic function of T-AS1 is dependent on its direct downstream effector MPZL1.

## Introduction

Gastric cancer (GC) is the fourth-leading cause of cancer-related mortality globally 1 and the third-leading cause in China 2. Since patients are often diagnosed at an advanced stage with limited therapeutic strategies, the 5-year overall survival rate of GC patients remains unsatisfactory 3. Although great advances have been made in GC treatment, the molecular mechanisms underlying its tumorigenesis and metastasis are still poorly understood.

Aberrant expression of long non-coding RNAs (lncRNA) have been reported to be involved in cell proliferation, tumor progression, invasion, and metastasis 4, 5. LncRNAs can act as enhancers, endogenous siRNA, scaffolds, or decoys through physical interaction with other RNA species or proteins, directly affecting cell-signaling cascades. Accumulating evidence shows that lncRNAs are involved in the pathogenesis and progression of many kinds of cancers 6 and play a role in the promotion or suppression of GC 7. lncRNAs also have diagnostic and prognostic potential and may even inform therapeutic options for cancer patients 8, 9.

We recently discovered that a novel lncRNA TNFRSF10A-AS1 (T-AS1) was overexpressed in GC 10. However, the functional significance and mechanisms of T-AS1 action in GC are largely unknown. This study was to investigate the functional role, molecular mechanism and clinical implication of T-AS1 in gastric carcinogenesis.

## Results

### T-AS1 is upregulated in GC cells and primary GC tumors

**Figure 1:**
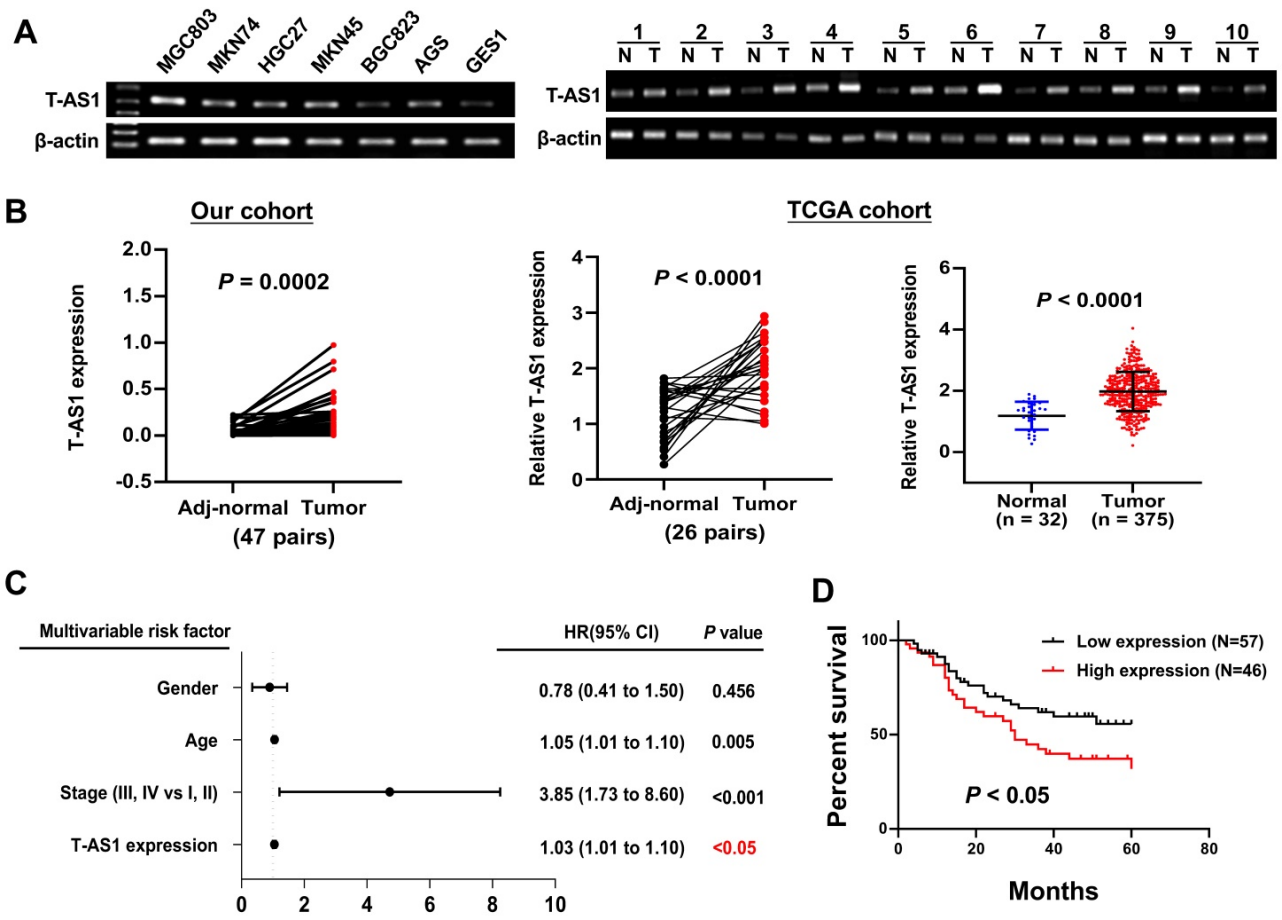
** T-AS1 was highly expressed and correlated with an increased risk of death in patients with gastric cancer. A,** T-AS1 was highly expressed in GC cell lines but not in the normal gastric cell line GES-1, as shown by RT-PCR. T-AS1 expression was upregulated in GC compared with paired adjacent normal tissues, as shown by RT-PCR. **B,** T-AS1 expression was upregulated in GC compared with paired adjacent normal tissues, as shown by qRT-PCR. RNA-seq data from the TCGA study also show upregulation of T-AS1 in GC as compared with adjacent normal tissues (bottom right, paired, and unpaired samples). **C,** Multivariable Cox analysis of T-AS1 in our cohort by forest plot. **D,** Kaplan-Meier survival analysis showed GC patients with high T-AS1 expression had poorer survival than those with low T-AS1 expression from our cohort.

T-AS1 expression in GC cells and primary GC tissues was determined by RT-PCR and qRT-PCR, T-AS1 was highly expressed in all 6 GC cell lines (MGC803, MKN74, HGC27, MKN45, BGC823 and AGS), but low expressed in the normal gastric cell GES1 (**Figure 1A**). Upregulation of T-AS1 in GC tumors was confirmed in two different cohorts. T-AS1 expression was significantly upregulated in primary gastric tumors as compared with adjacent normal tissues (n = 47) as determined by qRT-PCR (*P* < 0.001) in our cohort. Consistently, T-AS1 was upregulated in GC paired samples (n = 26, *P* < 0.0001) and unpaired samples (tumor, n = 375; normal, n = 32; *P* < 0.0001) from TCGA cohort (**Figure 1B**). These results demonstrated that T-AS1 was commonly overexpressed in GC.

### T-AS1 high expression is associated with the survival in patients with GC

We further evaluated the clinicopathologic feature and the mortality risk of T-AS1 expression in GC patients. In primary GC tissues from 103 Chinese patients (**Table S1**), multivariate Cox regression analysis showed that T-AS1 expression was an independent poor prognostic factor for GC patients (HR, 1.03;* P* = 0.011) (**Figure 1C**). Kaplan-Meier survival curves showed that GC patients with high T-AS1 expression had significantly shorter survival than those with low T-AS1 expression (*P* <0.05) (**Figure 1D**).

### TNFRSF10A-AS1 promotes GC cell growth* in vitro and in vivo*

**Figure 2:**
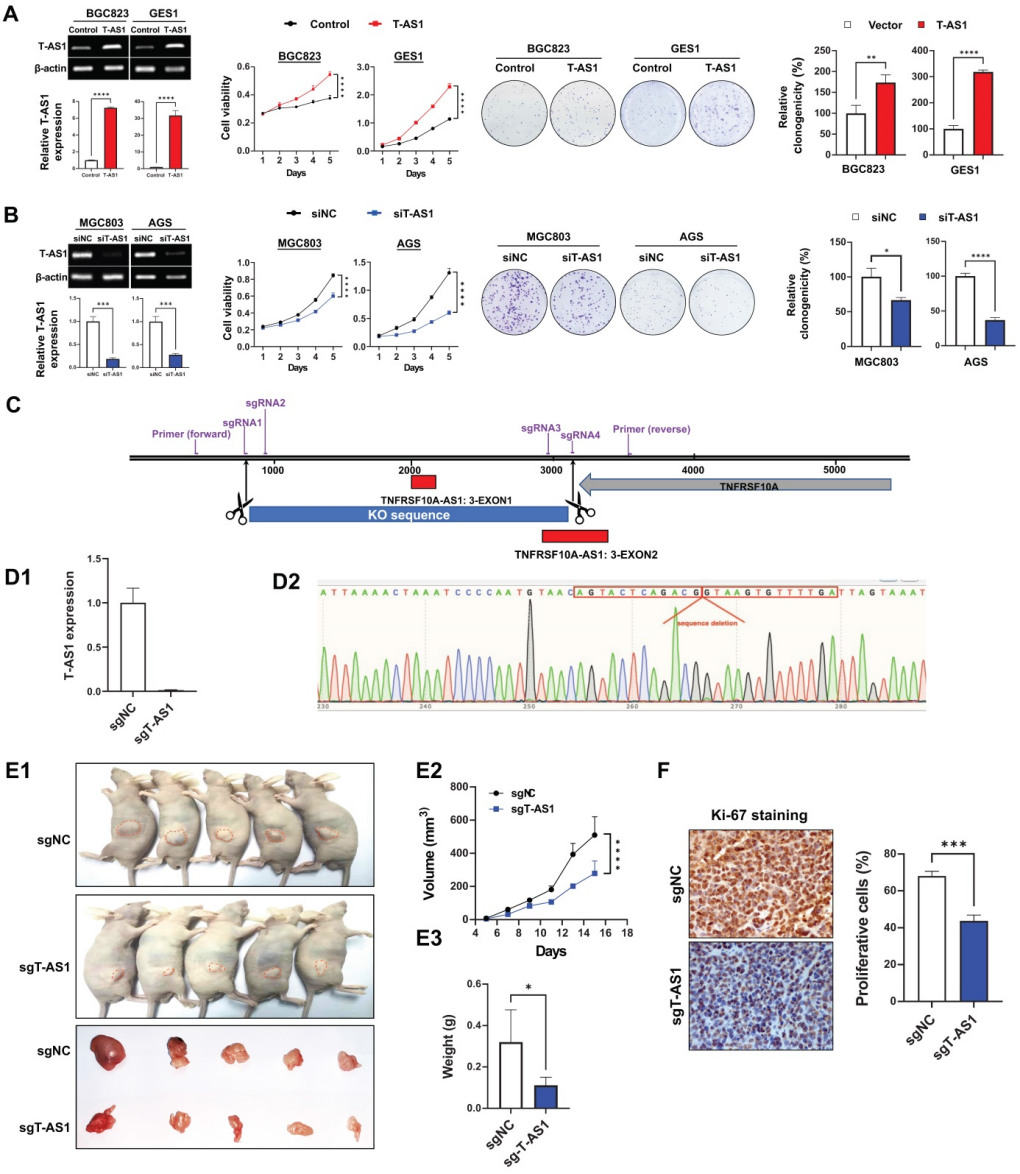
T-AS1 promotes cell proliferation *in vitro* and* in vivo*. **A,** Ectopic expression of T-AS1 in BGC823 and GES1 cells was confirmed by RT-PCR and qRT-PCR analysis, showing significantly increased cell viability and clonogenicity. **B,** Knockdown of T-AS1 by siT-AS1 in MGC803 and AGS was confirmed by RT-PCR and qRT-PCR analysis, indicating significant inhibition of cell viability and colony formation. **C,** T-AS1 knockout illustration by CRISPR/Cas9 assay. **D,** The T-AS1 sequence were knocked out in MGC803 cells. T-AS1 were knocked out through qRT-PCR (**D1**), and genome sequencing (**D2**). **E1,** Representative images of tumor growth in nude mice subcutaneously inoculated with sgNC or sgT-AS1 transfected MGC803 cells were shown. T-AS1 knockout inhibited subcutaneous tumorigenicity *in vivo,* as shown by the growth curve of tumor volume (**E2**) and weight (**E3**) at the end of the experiment. Data were expressed as mean ± SD, n = 10/group. **F,** Ki-67 protein expression in subcutaneous xenografts were determined by immunohistochemistry.

Overexpression were performed on BGC823 and GES1 cells which expressed T-AS1 at low level, and knockdown were performed on MGC803 and AGS cells which with high level of T-AS1 and more commonly used in gastric cancer research. *In vitro* gain- and loss-of-function assays of T-AS1 showed that ectopic expression of T-AS1 in BGC823 and GES1 cells significantly increased cell viability and clonogenicity (**Figure 2A**). Conversely, T-AS1 knockdown by RNA interference in MGC803 and AGS cells markedly inhibited cell viability and clonogenicity (**Figure 2B**). To investigate the effect of T-AS1 on subcutaneous xenograft tumors, CRISPR/Cas9 was used to knock out the T-AS1 exon in the gene and T-AS1 stable knockout was constructed in MGC803 cells. Four sgRNAs was used to knock out Exon 1 and part of Exon 2 of T-AS1 gene (**Figure 2C**). The knockout effect was confirmed by qRT-PCR and gene sequencing (*P* < 0.001, **Figure 2D**). We subcutaneously injected MGC803 cells stably knock out T-AS1-KO or NC into the left and right dorsal flanks of nude mice, respectively. As shown in **Figure 2E1**, knockout of T-AS1 markedly decreased the size (*P* < 0.0001) and weight (*P* < 0.05) of MGC803 xenograft tumors (**Figure 2E2 and 2E3**). Ki-67 staining further confirmed that knockout of T-AS1 inhibited cell proliferation evidenced by reduced Ki-67 positive cells (*P* < 0.001, **Figure 2F**).

### T-AS1 inhibits cell apoptosis by activating p53 signaling

**Figure 3:**
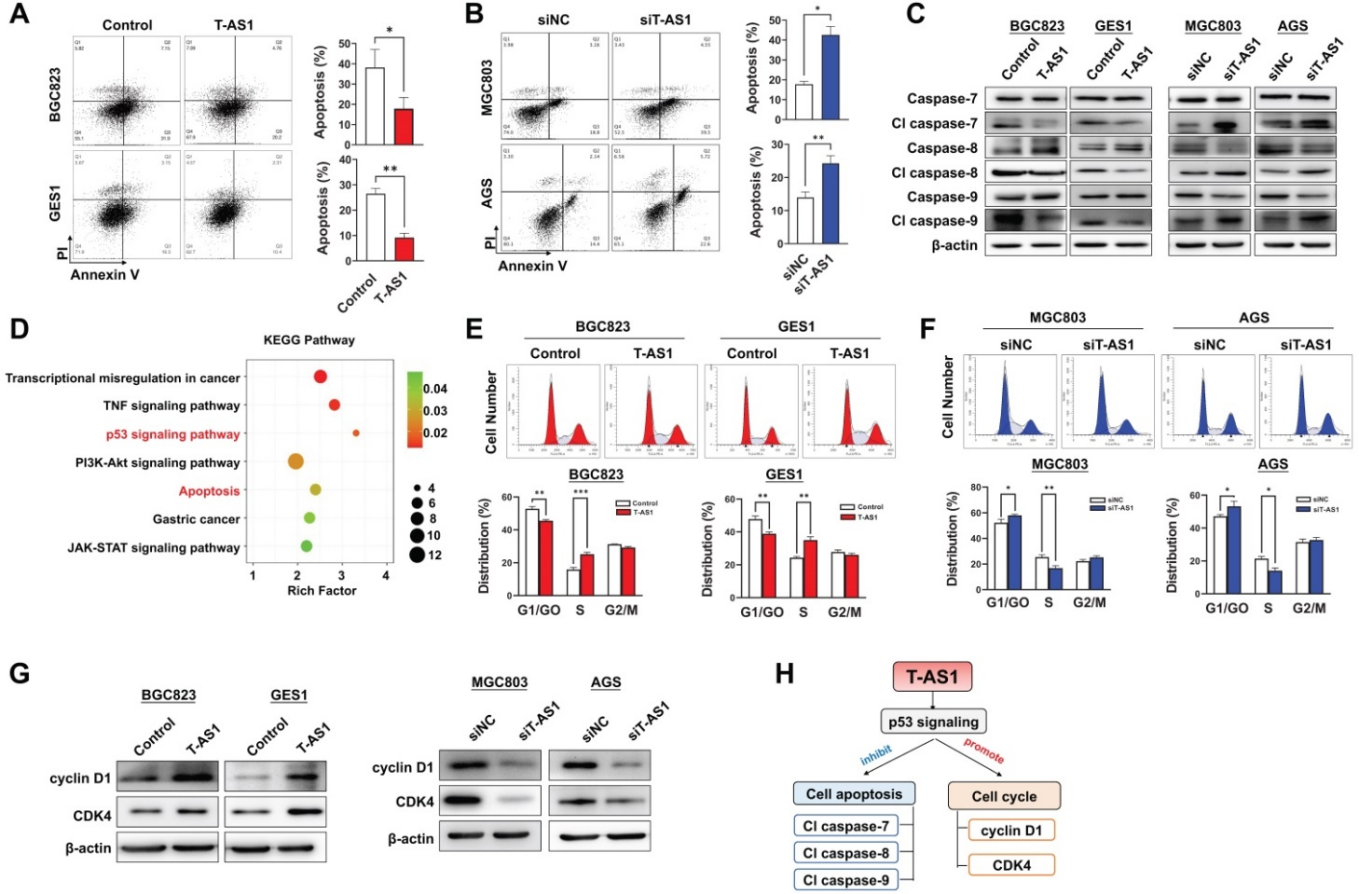
** T-AS1 inhibits gastric cell apoptosis and promotes cell cell-cycle progression. A,** Overexpression of T-AS1 inhibited cell apoptosis by flow cytometry after Annexin V/PI dual staining.** B,** Knockdown of T-AS1 promoted cell apoptosis. **C,** Western blot analysis showed T-AS1 expression reduced activation of caspase-7, caspase-8, caspase-9, while knockdown of T-AS1 exhibited the opposite effect. **D,** KEGG pathways enriched by differentially expressed genes affected by TNFRSF10A-AS1. **E,** Overexpression of T-AS1 significantly increased the number of cells in S-phase, while knockdown of T-AS1 had the opposite effect** (F)**. **G,** Western blot analysis showed T-AS1 increased the protein levels of cyclin D1 and CDK4, while T-AS1 knockdown had the opposite effect. **H,** Schematic illustration of the content of Figure 3.

Apoptosis analysis by flow cytometry showed that T-AS1 significant reduced total apoptotic cells in BGC823 and GES1 cells transfected with T-AS1 compared to the control cells (all *P* < 0.05) (**Figure 3A**), while knockdown of T-AS1 significantly increased apoptosis cells of MGC803 and AGS cells (both *P* < 0.05) (**Figure 3B**). The T-AS1 induced decrease in apoptosis was confirmed by the elevated protein expression of key apoptosis markers (cleaved forms of caspase-7, caspase-8, and caspase-9), while knockdown of T-AS1 showed the converse effects (**Figure 3C**).

To understand the molecular basis of the oncogenic property of T-AS1, RNA-sequencing was performed in T-AS1 knockdown and control GC cells (MGC803 and AGS). KEGG pathway enrichment analysis showed that knockdown of T-AS1 significantly activated tumor suppressive p53 signaling (**Figure 3D and Table S6**).

### T-AS1 promotes cell-cycle progression

We next investigated the effect of T-AS1 on cell cycle progression by flow cytometry. Ectopic expression of T-AS1 accelerated G1/S phase progression in BGC823 (*P* < 0.01) and GES1 cells (*P* < 0.01, **Figure 3E**). Conversely, knockdown of T-AS1 by RNA interference in MGC 803 and AGS cells decelerated the G1-S cell cycle transition (*P* < 0.05, **Figure 3F**). The enhanced G1/S cell cycle by T-AS1 was confirmed by the upregulation of the key G0/G1 phage regulators CDK4 and cyclin D1, and knockdown of T-AS1 showed opposite effect (**Figure 3G**). Our results indicated that T-AS1 could promote cell cycle and inhibit cell apoptosis by activating p53 signaling (**Figure 3H**).

### T-AS1 promotes cell migration and invasive abilities of GC cells

**Figure 4:**
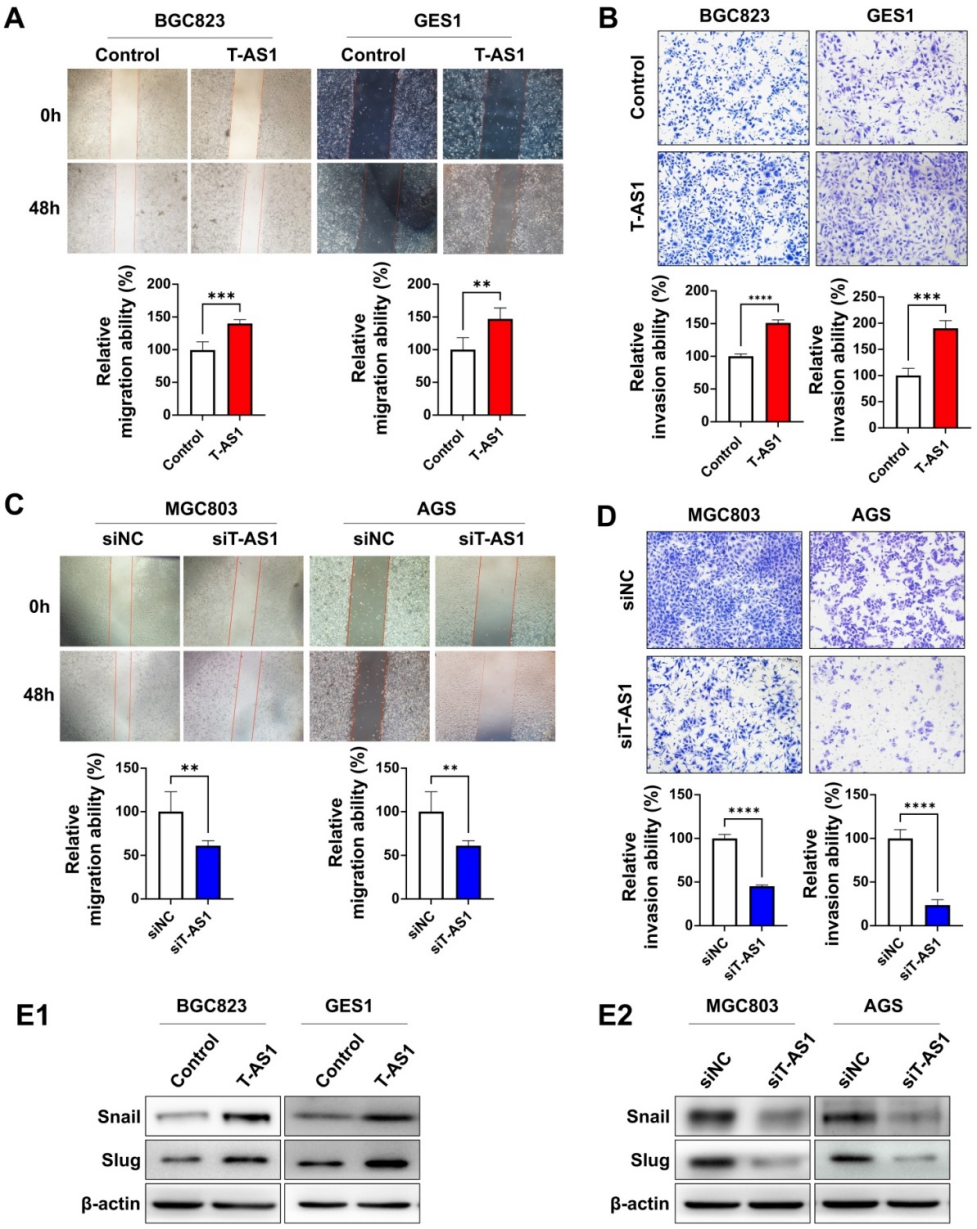
** T-AS1 promotes migration and invasion of GC cells. A,** Representative images of wound healing assay revealed that ectopic expression of T-AS1 promoted cell migration in BGC823 and GES1 cells. **B,** Representative images of Matrigel invasion trans well assay revealed that ectopic expression of T-AS1 promoted cell invasion in BGC823 and GES1 cells. **C,** Knockdown of T-AS1 significantly inhibited migration ability. **D,** Knockdown of T-AS1 significantly decreased cell invasion ability. **E,** T-AS1 expression increased the levels of Snail and Slug (**E1**), while knockdown of T-AS1 showed the opposite effect on these EMT markers (**E2**).

Wound-healing and Matrigel invasion assays were performed to determine the effects of T-AS1 on cell migration and invasion. Ectopic expression of T-AS1 significantly increased the migration and invasiveness abilities of BGC823 cells (all *P* < 0.001) and GES1 cells (all *P* < 0.01, **Figure 4A and 4B**). On the other hand, knockdown of T-AS1 markedly suppressed the migration and invasion abilities of MGC803 and AGS cells (**Figure 4C and 4D**). Protein expression of epithelial-to-mesenchymal transition markers was investigated by western blot. T-AS1 overexpression upregulated the expression of Snail and Slug (**Figure 4E1**), while knockdown of T-AS1 had the opposite effects (**Figure 4E2**). These findings demonstrated that the oncogenic effect of T-AS1 was also associated with its role in promoting cell migration and invasion of GC cells.

### MPZL1 is the direct downstream effector of T-AS1 in GC

**Figure 5:**
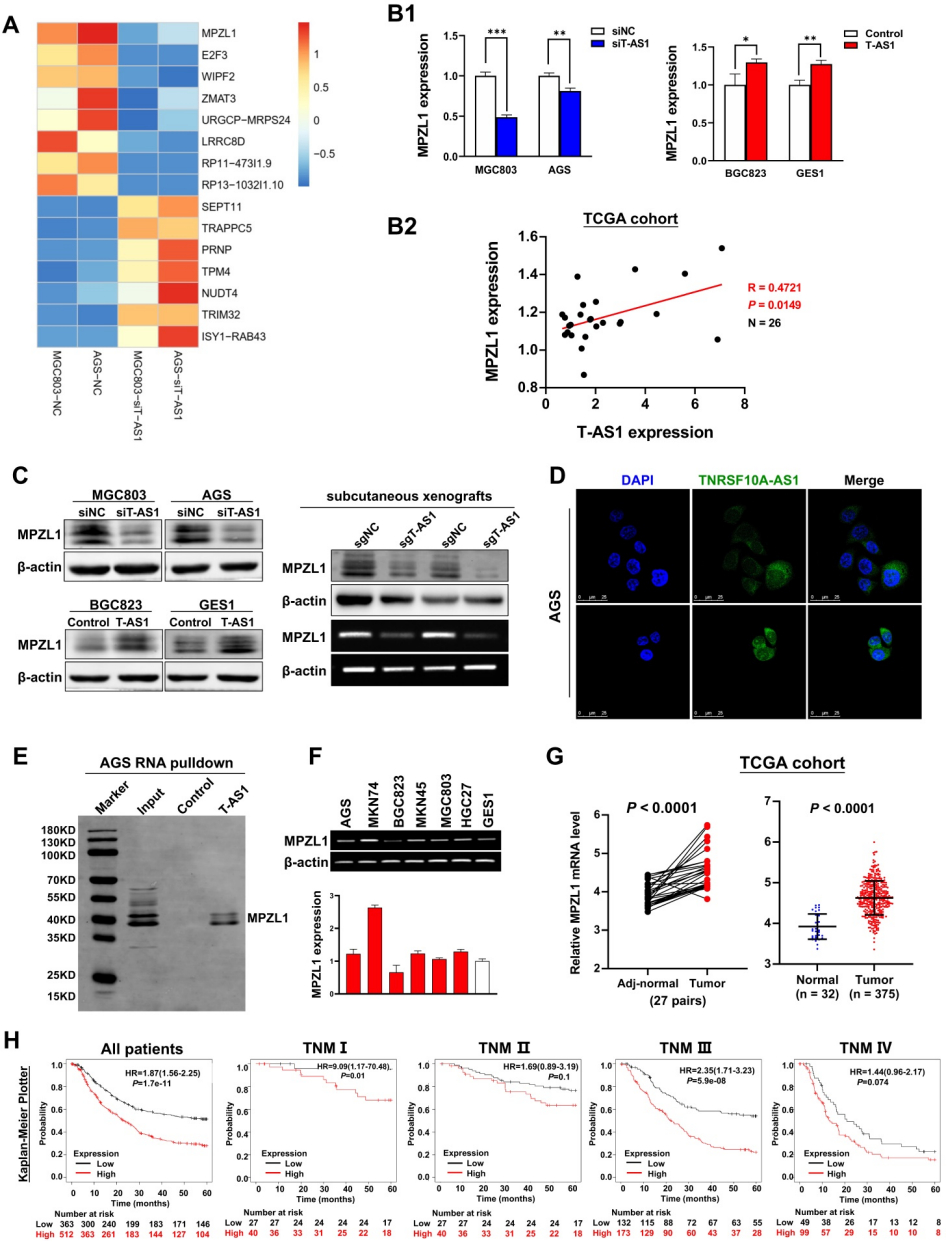
** MPZL1 was the downstream target of T-AS1. A,** Gene expression changes after knockdown of T-AS1 by RNA sequencing. **B,** MPZL1 mRNA expression was correlated with the T-AS1 expression. T-AS1 knockdown decreased the expression of MPZL1 in MGC803 and AGS cells, whereas ectopic expression of T-AS1 increased the mRNA expression of MPZL1 in BGC823 and GES1 by qRT-PCR **(B1)**. MPZL1 mRNA expression was positively correlated with the expression of TNFRSF10A-AS1 in the TCGA cohort **(B2)**. **C,** MPZL1 expression was correlated with the TNFRSF10A-AS1 expression by western blot in GC cells and in subcutaneous xenografts.** D,** RNA FISH results showed that T-AS1 was distributed in both cytoplasm and nucleus, but mainly in the cytoplasm. **E,** RNA pull-down showed that T-AS1 interacts with MPZL1 in AGS cell.** F,** MPZL1 was frequently upregulated in GC cell lines (AGS, MKN74, MKN45, MGC803, HGC27) as determined by qRT-PCR and RT-PCR.** G,** MPZL1 expression was increased in gastric tumor tissues from the TCGA cohort.** H,** Kaplan-Meier survival analysis in GC patients with different MPZL1 expression in TCGA dataset.

To elucidate the molecular mechanisms underlying the pro-tumorigenic action of T-AS1, gene expression profiles were analyzed by RNA sequencing in T-AS1 knockdown MGC803 and AGS cells. MPZL1 was the most significantly downregulated gene in T-AS1 knockdown cells (**Figure 5A**). T-AS1 expression was positively correlated with MPZL1 mRNA expression on cells (**Figure 5B1**) and in TCGA cohort (R=0.4721, *P* = 0.0149, **Figure 5B2**). Moreover, T-AS1 expression was also correlated with MPZL1 at protein level by Western blot (**Figure 5C**). lncLocator analysis showed that T-AS1 mainly located in cell cytoplasm (**Figure S1A**). RNA FISH analysis using eight probes of T-AS1 gene (**Table S2**) confirmed that T-AS1 was mainly located in the cytoplasm in AGS cells and MGC803 cells (**Figure 5D and Figure S1B**). RNA pull-down assays confirmed that T-AS1 could bind to MPZL1 (**Figure 5E**).

### MPZL1 shows oncogenic function in GC and is associated with poor survival of GC patients

MPZL1 mRNA expression was upregulated in 5 GC cell lines (AGS, MKN74, MKN45, MGC803, HGC27) but not in BGC823 and GES1 cell lines (**Figure 5F**). MPZL1 mRNA expression was significantly higher in GC tumor tissues compared to adjacent normal tissues of paired samples (n = 27, *P* < 0.0001); and in GC tumor tissues compared to normal gastric tissues of unpaired samples (Normal, n = 32; Tumor, n = 375; *P* < 0.0001) (**Figure 5G**). Kaplan-Meier analysis showed that high MPZL1 expression was correlated with poor overall survival of GC patients (**Figure 5H**). To investigate the function of MPZL1 in GC, knockdown of MPZL1 by RNA interference in MGC803 and AGS cells, low expression of MPZL1 was confirmed by qRT-PCR and western blot (**Figure S2A**). Moreover, MPZL1 expression promoted cell proliferation and clonogenicity in MGC803 and AGS cells (**Figure S2B**). MPZL1 also promoted migration and invasion in MGC803 and AGS cells (**Figure S2C**).

### The oncogenic role of T-AS1 is partially dependent on MPZL1

**Figure 6:**
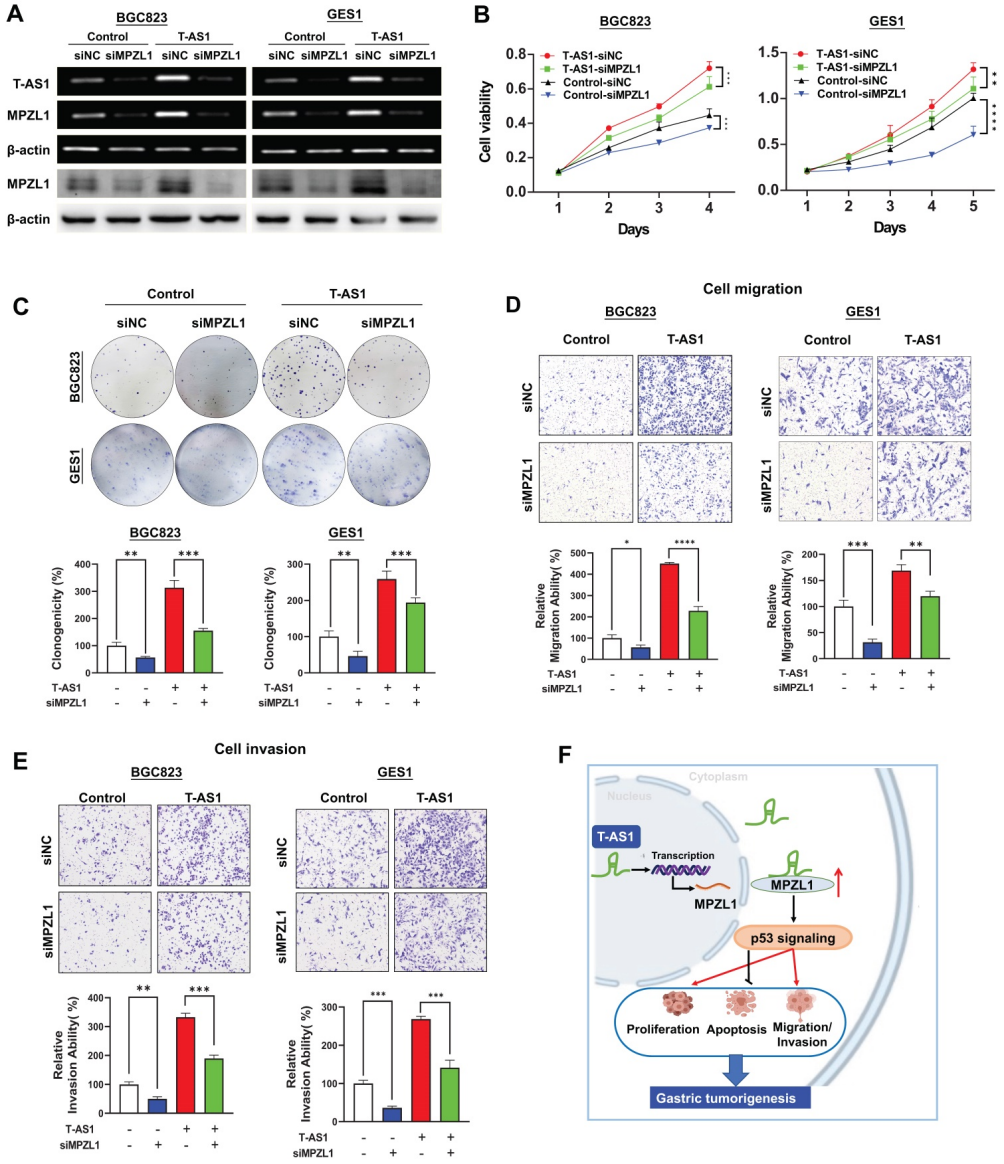
** T-AS1 exerts tumor-promoting function partially depending on the tumor-promoting MPZL1. A,** Successful knockdown of MPZL1 in T-AS1 overexpressed cells was confirmed by RT-PCR and western blot analysis. **B,** Effect of T-AS1 on cell viability with or without MPZL1 knockdown by MTT assay was detected. Data are mean ± SD (right). **, *P* < 0.01; ***,* P* < 0.001; ****, *P* < 0.0001.** C,** Effect of T-AS1 on colony formation ability with or without MPZL1 knockdown was investigated. MPZL1 knockdown partially abolished the tumor-promoting function of T-AS1. MPZL1 knockdown partially abolished the migration (**D**) and invasion (**E**) ability of T-AS1 in BGC823 and GES1 cells. **F,** Schematic illustration of the molecular mechanism of T-AS1 in gastric cancer.

We then assessed whether the oncogenic function of T-AS1 in GC was dependent on MPZL1. BGC823 and GES1 cells stably transfected with T-AS1 or control vector were co-transfected with siRNA against MPZL1 (**Figure 6A**). MPZL1 knockdown in BGC823 and GES1 cells significantly abolished the promoting effect of T-AS1 on cell viability **(***P* < 0.001, **Figure 6B**) and clonogenicity (*P* < 0.001, **Figure 6C**). Knockdown of MPZL1 significantly blunted the promoting effects of T-AS1 overexpression on cell migration and invasion in BGC823 and GES1 cells (all *P* < 0.01, **Figure 6D and 6E**). These results indicated that the tumor-promoting effect of T-AS1 is at least in part dependent on MPZL1 in GC.

## Discussion

In this study, we found that T-AS1 was highly expressed in GC tumors tissues and in GC cell lines. We investigated the clinical implication of T-AS1 expression in GC and found that T-AS1 expression was an independent poor prognostic factor for GC patients and correlated with an increased risk of death. Therefore, T-AS1 might serve as a prognostic biomarker in GC patients.

In this connection, we investigated the functional significance of T-AS1 in GC both *in vitro* and *in vivo*. *In vitro* gain- and loss-of-function assays showed that overexpression of T-AS1 in BGC823 and GES1 cells significantly increased cell viability and colony formation ability. Overexpression of T-AS1 also promoted the G1/S cell cycle transition by upregulating of cyclin D1 and CDK4 in CRC cell lines concomitant with inhibition of p53 pathway. T-AS1 also significantly increased cell migration and invasion abilities, but suppressed cell apoptosis. Conversely, T-AS1 knockdown in MGC803 and AGS cells significantly suppressed cell growth, migration/invasion, but induced cell apoptosis. We furtherly generated stalely T-AS1 knockout MGC803 cells, T-AS1 knockout in MGC803 cells inhibited tumor growth in mouse subcutaneous xenograft models *in vitro* and decreased the expression of proliferation-associated marker Ki-67. These findings collectively indicated that T-AS1 plays an oncogenic function in GC through modulating cell proliferation, cell cycle, apoptosis and migration/invasion.

The molecular mechanisms by which T-AS1 exerts its pro-tumorigenic and pro-metastatic functions in GC were evaluated by RNA sequencing in T-AS1 knockdown and control GC cells. RNA sequencing analysis showed that MPZL1 is one of the critical downstream targets of T-AS1. T-AS1 expression correlated positively with MPZL1 from GC cells and GC tissues. RNA pull-down assay confirmed T-AS1 interacted with MPZL1. Since T-AS1 localized both in cell cytoplasm and nucleus, we hypothesis that T-AS1 may be involved in transcriptional and post-transcriptional regulatory processes to alter the expression and function of downstream targets MPZL1.

MPZL1 was reported to promote tumor growth in several cancer types 11, 12. We found that MPZL1 was upregulated in GC cells. High expression of MPZL1 was associated with poor prognosis in GC. In keeping with our finding, data from the Human Protein Atlas showed that MPZL1 overexpression was associated with a poor prognosis in renal, liver, lung and thyroid cancers (www.proteinatlas.org/ENSG00000197965-MPZL1/pathology). We then assessed whether the tumor-promoting function of T-AS1 in GC depends on MPZL1. The rescue experiment showed that knockdown of MPZL1 by siRNA in BGC823 and GES1 cells significantly blunted the promoting effects of T-AS1 on cell verbality, colony formation, cell migration/invasion abilities, inferring that the tumor-promoting effect of T-AS1 is at least in part dependent on MPZL1 in GC.

In conclusion, our study showed for the first time that lncRNA T-AS1 is a tumor-promoting factor in GC through inducing cell proliferation, cell-cycle transition, metastasis/invasion, but inhibiting apoptosis. T-AS1 directly binds to its downstream effector MPZL1 to induce its expression, an oncogenic factor. The tumor promoting function of T-AS1 is at least in part depending on MPZL1 expression **(Figure 6F)**. Moreover, T-AS1 may service as a prognostic factor for GC patients.

## Materials and Methods

### Patients and human samples

The study cohort included 103 patients with histologically confirmed GC at Peking University Cancer Hospital, Beijing, China, including 47 paired GC tumor tissues and adjacent nontumor tissues. Detailed patient information is shown in **Table S1**. The tumor-node-metastasis stage of GC was determined according to the classifications recommended by the American Joint Committee on Cancer, 7th edition. All subjects provided written informed consent for obtaining the study specimens. This study was approved by the Institutional Review Boards of Peking University Cancer Hospital and the Clinical Research Ethics Committee of the Chinese University of Hong Kong. This study was carried out in accordance with the Declaration of Helsinki of the World Medical Association.

### Cell lines

Six GC cell lines (AGS, BGC823, MKN74, MGC803, HGC27, and MKN45) and one normal gastric epithelial cell line GES1 were used in this study. AGS was obtained from the ATCC. BGC823, MGC803, HGC27 and GES1 were obtained from Cell Research Institute, Shanghai, China. MKN74 was obtained from the Japanese Collection of Research Bioresources Cell Bank. These cell lines were obtained between 2014 and 2015, and cell authentication of cells we performed the function and mechanisms study (AGS, BGC823, MGC803 and GES1) was verified by short tandem repeat profiling. Cells were cultured in DMEM (Gibco BRL, supplemented with 10% FBS). Routine mycoplasma testing was performed by PCR. Cells were grown for no more than 20 passages in total for any experiment.

### *In vivo* tumorigenicity assays

To assess the effect of T-AS1 on tumor growth, MGC803 cells (5 × 10^6^ cells) stably transfected with sgNC or sgT-AS1 were injected subcutaneously into the left and right flanks of 4-week-old male Balb/c nude mice (n = 10 per group). Tumor volume (mm^3^) was calculated using the longest and shortest diameters of the tumor as previously described 13. At the end of the experiments, the mice were sacrificed and the tumors were weighed and stored for further analysis. All animal experimental procedures were approved by the Animal Ethics Committee of the Chinese University of Hong Kong.

### Fluorescence *in situ* hybridization (FISH)

T-AS1 expression in GC cells was detected using Stellaris RNA FISH with 8 independent probes (Bio search Technologies, Hoddesdon, UK) on cell slides according to the protocol provided by the manufacturer. Probe sequences are listed in **Table S2**. Briefly, cell slides were fixed in 4% paraformaldehyde and hybridized with T-AS1 probes (200 nM) for 16h at 37 °C. Chromosomes were stained with DAPI in PBS for 5 min. Immunofluorescence images were taken with a fluorescence microscope (Olympus, Tokyo, Japan).

### RNA sequencing

A 3-μg sample of RNA was used as the input material for RNA sample preparation. All samples had RNA integrity values > 6.8. Sequencing libraries were generated using the IlluminaTruSeq RNA Sample Preparation Kit (Illumina Inc., San Diego, CA, USA) following the manufacturer's recommendations. The libraries were sequenced using the Illumina HiSeq X-ten platform as per the manufacturer's instructions (Shanghai Biotechnology Corp., Shanghai, China).

### RNA pull-down assay

Full-length T-AS1 was transcribed using T7 MEGAscript kits (Shanghai GenePharma, Shanghai, China) and labelled *in vitro* using the Pierce RNA 3′ End Desthiobiotinylation Kit (Thermo Fisher Scientific, Waltham, MA, USA). RNA pull-down assays were performed using the Pierce Magnetic RNA-Protein Pull-Down Kit (Thermo Fisher Scientific). Desthiobiotin-labelled RNA (50 pM) was mixed with 50 μL of magnetic beads and incubated with AGS cell protein lysates for 60 min at 4 °C with rotation. The beads were washed briefly 4 times, boiled in SDS buffer and the RNA-binding proteins were detected by western blot.

### CRISPR/Cas9 knockout assay

The lentiviral sgT-AS1 construct, as reported previously 14 (Genechem Technologies, Shanghai, China), was transfected into MGC803 cells using the vector CV279 MCS-EF1a-Cas9-FLAG-P2A-puro. The T-AS1 sequence and the sgRNA position of the 4 sgRNAs used in this assay are shown in **Table S3**. After transfection, MGC803 cells were selected by puromycin and distributed into 192-well plates. sgT-AS1 in single stable cells was verified by PCR using the detection primer shown in **Table S4**. Control cells were treated with an empty vector containing nonsense sgRNA.

### Statistical analysis

Data are expressed as the mean ± SD. Statistical analysis was performed using the Statistical Package for the Social Sciences (standard V.22.0; IBM Corporation, A). The χ^2^ test was used to compare patient characteristics and determine distributions of expression and covariates by vital status. The Mann-Whitney U test or Student's t test was used to compare variables between two groups. The cutoff value was determined by survival significance analysis using the tool Cutoff Finder (http://molpath.charite.de/cutoff/) 15. Overall survival in relation to expression was evaluated by the Kaplan-Meier survival curve and the log rank test. The difference in cell viability and tumor growth rate between the two groups was determined by repeated-measures analysis of variance. *P* < 0.05 was considered statistically significant, and all tests were two-tailed.

## Supplementary Material

Supplementary methods, figures and tables.

## Abbreviations

GC: gastric cancer
T-AS1: TNFRSF10A-AS1
MPZL1: Myelin protein zero like 1
TCGA: The Cancer Genome Atlas
TNM: tumor nodes-metastasis
IHC: immunohistochemical
RR: relative risk
EMT: epithelial-mesenchymal transition
FISH: Fluorescence *in situ* hybridization
CRISPR: Clustered regularly interspaced short palindromic repeats
