# Supplementary material for: LncRNA TNFRSF10A-AS1 promotes gastric cancer by directly binding to oncogenic MPZL1 and is associated with patient outcome
Source: Int J Biol Sci. 2022 May 1;18(8):3156–66. doi: 10.7150/ijbs.68776 (PMC9134913; doi:10.7150/ijbs.68776)
Supplement: Supplementary file 1 — Supplementary methods, figures and tables. [file ijbsv18p3156s1.pdf]

**A**

--------lncLocator Prediction Result --------

| Subcellular locations | score |
|---|---|
| Cytoplasm | 0.691117546327 |
| Nucleus | 0.164720143282 |
| Ribosome | 0.0237790882526 |
| Cytosol | 0.0461992240767 |
| Exosome | 0.0741839980614 |

| Predicted location |
|---|
| Cytoplasm |

**B**

DAPI TNRSF10A-AS1 Merge

MGC803

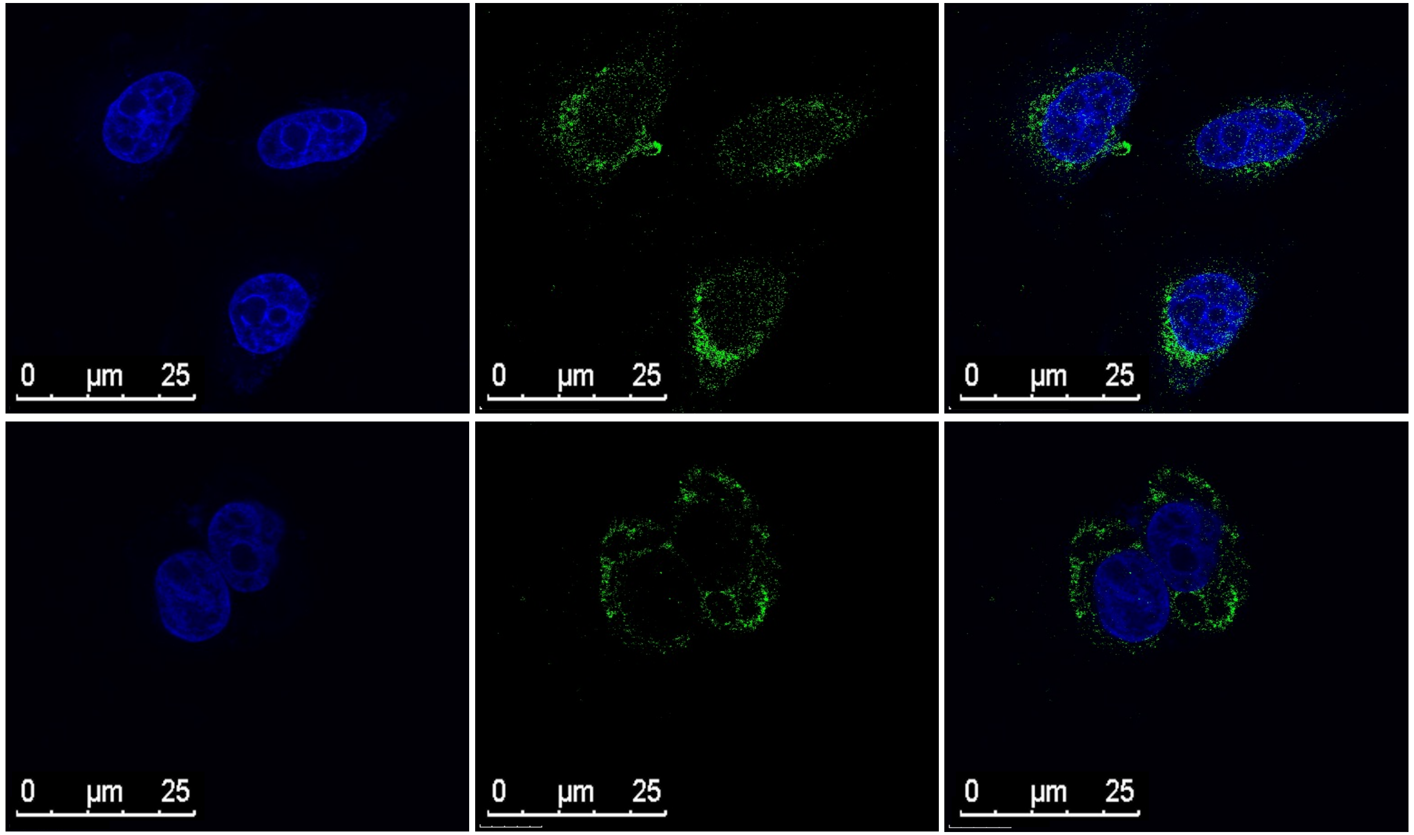


**Supplement Figure 1. The subcellular localization of TNFRSF10A-AS1. A,** TNFRSF10A-AS1 was mainly distributed in the cytoplasm via the application site (http://www.csbio.sjtu.edu.cn/bioinf/lncLocator/index.html). **B,** RNA FISH results showed that TNFRSF10A-AS1 was distributed in both cytoplasm and nucleus, but mainly in the cytoplasm in MGC803 cell.

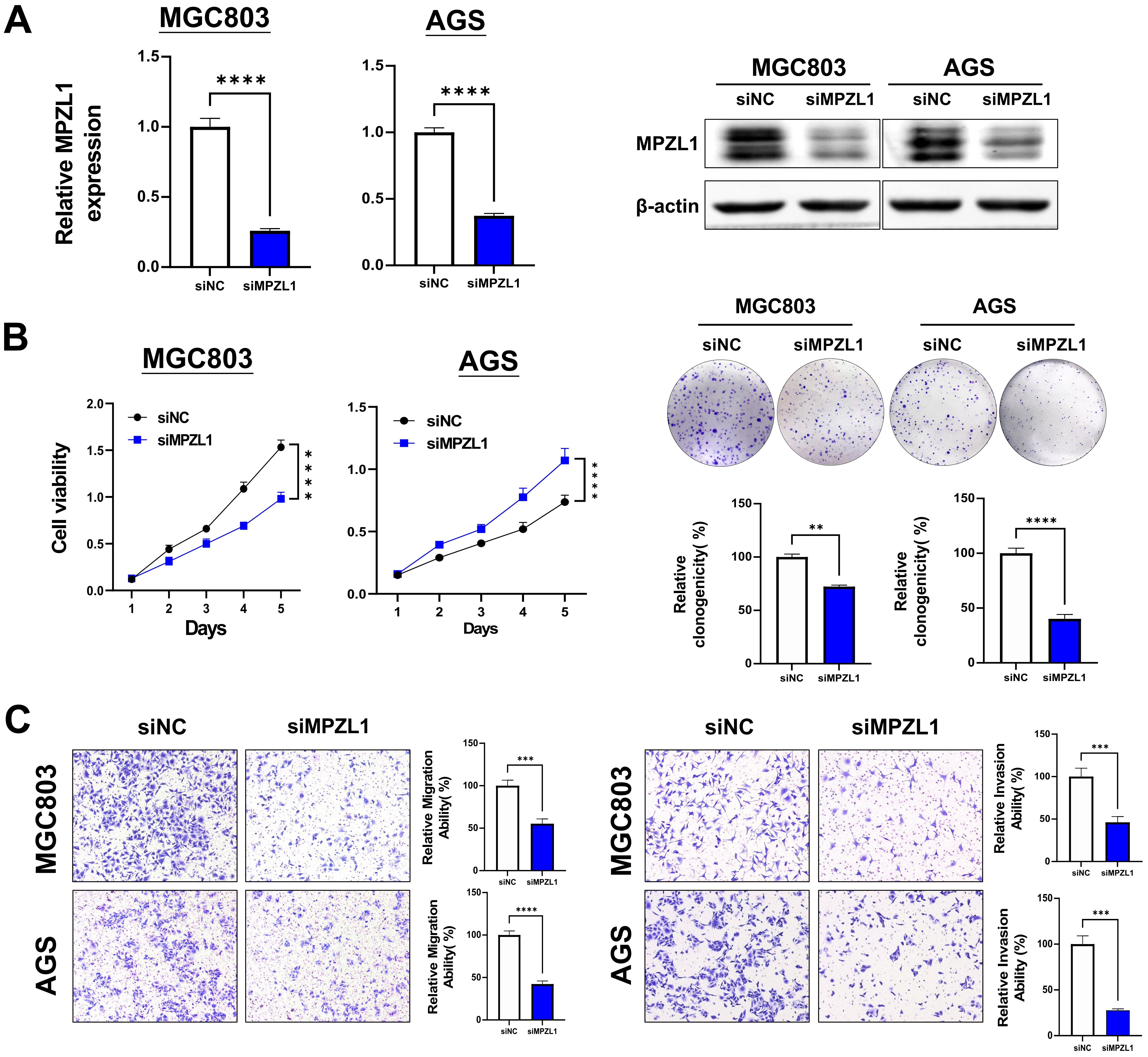


**Supplementary Figure 2.** MPZL1 showed oncogenic function in gastric cancer. **A,** Knockdown of MPZL1 in MGC803 and AGS cells was confirmed by qRT-PCR and western blot analysis. **B,** Knockdown of MPZL1 significantly inhibited cell viability (left panel) and colony formation ability (right panel) in MGC803 and AGS cells. **C,** Knockdown of MPZL1 expression inhibited cell migration (left panel) and cell invasion (right panel).

**Supplementary methods**

**RNA interference**

TNFRSF10A-AS1 siRNA (siTNFRSF10A-AS1: sense: 5'-CCAGACAGAUGGAUACCAATT-3'; antisense: 5'-UACCUAUGCAAAGAUUUGGTT-3'), MPZL1 siRNA (siMPZL1: sense: 5'-UCAAGUGGCAUAGCCAAUGTT-3'; antisense: 5'-CAUUGGCUAUGCCACUUGATT-3') and Negative Control (siNC) were ordered form Genepharma Company. 50 nmol of siMPZL1, siTNFRSF10A-AS1 or siNC were transfected into cells using Lipofectamine 2000 (Invitrogen) according to the manufacturer's instructions.

**RNA extraction, semi-quantitative RT-PCR, and real-time PCR analyses**

Total RNA was extracted from cells and tissues using TRIzol Reagent (Invitrogen). The extracted RNA was reversely transcribed into complementary DNA (cDNA) through a cDNA Reverse Transcription Kit (TransGen Biotech, Beijing). Semi-quantitative PCR was performed by AmpliTaq Gold DNA polymerase (Applied Biosystems; Thermo Fisher Scientific). Quantitative real-time PCR was performed by SYBR Green PCR Master Mix (Takara) on 7500HT Fast Real-Time PCR System (Applied Biosystems; Thermo Fisher Scientific). Experiments were repeated twice. actin was tested for normalization. Each sample was tested in triplicate. The 2-ΔΔCt method was employed to quantify the relative gene expression levels. The sequences of primers used are listed

in Supplementary Table S4.

**Protein extraction and Western blotting**

Total Protein was extracted from cells using RIPA lysis buffer. Total protein extracted from tissues were using T-PER Tissue Extraction Reagent I (Applied Biosystems; Thermo Fisher Scientific). Proteins were separated on SDS-polyacrylamide gel electrophoresis and transferred onto nitrocellulose membranes (GE Healthcare, Piscataway, NJ). Blots were immunostained with primary antibody and secondary antibody. Independent experiments were performed at least twice. The antibodies used are listed in Supplementary Table S5.

**Colony formation and cell growth curve assays**

Cells were plated in 6-well plates at 1,000 cells per well in complete DMEM. Medium was changed every 3 to 4 days. At the endpoint, cells were stained with 0.1% Crystal violet and the number of colonies consisting of >50 cells were counted. Cell growth curve was performed using 3-(4,5-dimethylthiazol-2-yl)-2,5-diphenyltetrazolium bromide (MTT) assay (Sigma-Aldrich).

**Immunocytochemistry staining**

Paraffin slides from xenograft were used. Ki-67 signal was assessed. The proliferation index was determined by counting the numbers of positive staining

cells of Ki-67 as percentages of the total number of colon cells. At least 1000 cells were counted each time.

**Migration and invasion assays**

For the “Transwell” migration assay, $3\times10^4$ cells with applied genetic modification in 200 μL serum-free medium were seeded onto the upper chamber of an 8-μm Transwell filter (Corning, 3422, Shanghai, China). In the lower chamber, 600 μL complete medium containing 20% FBS was added. After incubation, cells in the lower surface were fixed by methanol, stained with 1% crystal violet, and visualized under a microscope. For the “Transwell” invasion assays, matrigel-coated chambers (Corning, 354480, Shanghai, China) were used. For all “Transwell” assays in this study, five random views were included to calculate the average number of migrated/invaded cells.

**Apoptosis analyses**

Cells were plated in 12-well plates and serum-starved overnight. Annexin V Apoptosis Detection Kit APC (Invitrogen, Thermo Fisher) was used to determine cell apoptosis. The experiments were conducted three times in triplicates.

**Cell cycle analysis**

BGC823 and GES1 cells that were stably transfected with TNFRSF10A-AS1 or

empty vector were plated in a 6-well plate, while MGC803 and AGS cells were transfected with siTNFRSF10A-AS1 or siNC. After 48h of transfection, the cells were fixed in ice-cold 70% ethanol for 24h before staining with 50μg/ml propidium iodide (BD Biosciences, Franklin Lakes, NJ). The cells were sorted by BD AccuriTM C6 (BD Biosciences), and cell cycle distributions were analyzed using the ModFitLT 5.0 software (Verity Software House, Topsham, ME). All experiments were conducted three times in triplicates.

**Wound-healing assay**

Confluent cultures in 6-well plates were scratched with sterile P-200 pipette tips, washed, and cultured in DMEM containing 2% FBS. Cells were photographed after 0, 24, and 48 hours, respectively. The cells migrated across the gap wound were observed and documented using an inverted microscope. Distance of the gap was quantified using Image J.

**Ectopic overexpression of TNFRSF10A-AS1**

Overexpression of TNFRSF10A-AS1 in BGC823 or GES1 cells were performed by lentivirus mediated transfection. GC cells were transfected with a EF-1aF/GFP&Puro-TNFRSF10A-AS1 cDNA lentiviral vector ("TNFRSF10A-AS1 vector", Genepharma Company). The EF-1aF/GFP&Puro-mock lentiviral vector ("Empty vector") were utilized as controls. Vectors were first transfected to HEK-293T cells along with the lentivirus package plasmid mix to generate lentivirus.

At $6\times10^4$ cells per well, GC cells were initially seeded into six-well plates in polybrene-containing complete medium. The lentivirus was added to GC cells for 48h. Afterwards, cells were cultured in 1 puromycin (3 μg/mL)-containing medium for five more passages (10-12 days). In stable cells, expression of targeted gene was assessed by qRT-PCR.

## Supplementary Table 1. Clinical and pathological characteristics of patients in our cohort

| TNFRSF10A-AS1 expression (2^-∆ct) | Patient ID | Survival | Length | Sexy | Age | Location | TNM（7th） |
|---|---|---|---|---|---|---|---|
| 0.71233216 | 251-9536376T | 1 | 13 | Femal | 66 | L | 3c |
| 0.007920765 | 455-9541997T | 0 | 54 | Male | 57 | L | 2b |
| 0.03076696 | 481-9542761T | 1 | 20 | Male | 74 | M | 3b |
| 0.002458489 | 467-9542415T | 1 | 13 | Femal | 65 | U | 4 |
| 1.18609E-05 | 17-A25-20597T | 0 | 60 | Femal | 61 | M | 1b |
| 0.025441023 | 579-9547022T | 0 | 47 | Male | 67 | U | 2b |
| 0.013013894 | 573-9546266T | 1 | 31 | Male | 62 | M | 3c |
| 0.008064992 | 423-9541535T | 1 | 15 | Male | 63 | M | 3c |
| 0.001963502 | 187-Z11-24811T | 1 | 22 | Male | 68 | L | 2a |
| 5.05225E-05 | 167-A41-23993T | 0 | 25 | Male | 66 | U | 2a |
| 0.002565167 | 57-Z85-28072T | 0 | 58 | Male | 74 | L | 1b |
| 0.00585261 | 517-9544005T | 1 | 4 | Male | 69 | U | 3a |
| 0.001473082 | 411-9540961T | 0 | 56 | Femal | 59 | U | 3a |
| 0.006408494 | 511-9544004T | 1 | 29 | Male | 59 | U | 3c |
| 0.00527508 | 585-9547182T | 1 | 27 | Male | 64 | M | 3a |
| 0.004138951 | 525-9544427T | 0 | 50 | Femal | 71 | L | 2b |
| 0.01050092 | 211-A59-25914T | 0 | 7 | Male | 55 | L | 3c |
| 0.023810006 | 503-9532669T | 1 | 14 | Male | 56 | L | 4 |
| 0.027349625 | 513-9544112T | 0 | 51 | Male | 44 | U | 3b |
| 0.00302101 | 165-Z17-24876T | 0 | 48 | Male | 69 | M | 3b |
| 0.05143652 | 151-N9-18577T | 1 | 13 | Male | 56 | U | 3c |
| 0.003857332 | 13-Z123-28886T | 1 | 40 | Femal | 47 | L | 3c |
| 0.016114873 | 389-9540514T | 0 | 25 | Male | 44 | L | 2b |
| 0.001024175 | 51-Z159-33265T | 0 | 49 | Femal | 49 | M | 3a |
| 0.086496998 | 559-9546067T | 1 | 2 | Male | 71 | L | 4 |
| 0.02844763 | 171-N35-23553T | 1 | 22 | Male | 57 | U | 3a |
| 0.002799603 | 37-Z111-28681T | 0 | 49 | Male | 59 | L | 3c |

| | | | | | | | |
|---|---|---|---|---|---|---|---|
| 0.003689577 | 121-Z67-26306T | 0 | 60 | Male | 68 | L | 3b |
| 0.017013724 | 369-9539828T | 1 | 18 | Male | 58 | U | 2a |
| 0.100726333 | 19-Z153-29967T | 1 | 33 | Male | 78 | L | 3a |
| 0.001595925 | 469-9542538T | 0 | 52 | Femal | 74 | L | 3a |
| 0.00040513 | 179-Z51-26028T | 1 | 5 | Femal | 65 | L | 4 |
| 0.026322415 | 535-9544115T | 1 | 17 | Male | 66 | L | 3c |
| 0.000473474 | 207-A45-24664T | 0 | 38 | Male | 47 | U | 3c |
| 0.108625435 | 509-9532869T | 1 | 3 | Femal | 42 | M | 4 |
| 0.011326731 | 1-Z131-29411T | 0 | 46 | Femal | 40 | M | 2b |
| 0.008321937 | 431-9540474T | 1 | 6 | Male | 63 | U | 3a |
| 0.19688877 | 567-9546124T | 1 | 29 | Male | 60 | M | 2a |
| 0.00132209 | 169-N33-22820T | 1 | 10 | Male | 60 | L | 2a |
| 0.01354818 | 3-A79-26973T | 0 | 36 | Male | 42 | U | 3c |
| 0.006648973 | 117-Z21-24908T | 0 | 60 | Femal | 60 | L | 3c |
| 0.002131923 | 143-A35-23632T | 0 | 49 | Femal | 71 | M | 2b |
| 0.000470927 | 191-Z9-24758T | 1 | 12 | Male | 60 | L | 3b |
| 0.46755493 | 157-Z57-26261T | 0 | 60 | Femal | 67 | L | 2b |
| 0.022723862 | 501-9532666T | 1 | 44 | Femal | 50 | L | 3c |
| 0.004631928 | 43-Z129-28993T | 1 | 16 | Male | 64 | M | 3c |
| 0.022605443 | 395-9540809T | 0 | 37 | Male | 60 | L | 3c |
| 0.002160605 | 123-Z47-25943T | 0 | 60 | Femal | 38 | M | 3a |
| 0.004562804 | 435-9541634T | 0 | 17 | Male | 54 | U | 3b |
| 3.545386701 | 7-A17-19375T | 0 | 60 | Male | 37 | L | 3a |
| 0.01170474 | 447-9541880T | 0 | 50 | Male | 69 | L | 3c |
| 0.001784222 | 15-A21-19605T | 0 | 8 | Femal | 79 | U | 3b |
| 0.413159716 | 147-A51-25739T | 0 | 22 | Male | 67 | M | 3a |
| 0.006891285 | 85-A69-26043T | 0 | 44 | Male | 64 | U | 3b |
| 0.012235774 | 265-9537441T | 0 | 60 | Male | 64 | L | 2b |

| | | | | | | | |
|---|---|---|---|---|---|---|---|
| 0.001257128 | 79-Z105-28485T | 0 | 50 | Male | 61 | L | 3c |
| 0.014282986 | 415-9541385T | 1 | 15 | Male | 56 | U | 3b |
| 0.00724044 | 111-Z77-26914T | 1 | 51 | Male | 75 | L | 2a |
| 0.014634716 | 485-9542902T | 0 | 13 | Male | 49 | M | 1b |
| 0.0763574 | 105-Z25-25564T | 1 | 9 | Male | 75 | M | 3c |
| 0.394997114 | 87-N29-22640T | 1 | 27 | Femal | 69 | M | 3a |
| 0.01557702 | 119-Z71-26367T | 0 | 60 | Male | 47 | L | 2a |
| 0.00245678 | 67-Z133-29445T | 1 | 13 | Male | 66 | L | 4 |
| 0.001274167 | 203-N1-15388T | 1 | 23 | Femal | 63 | U | 3a |
| 0.05408886 | 349-9539309T | 0 | 13 | Male | 49 | U | 3a |
| 0.064423452 | 27-A27-21002T | 1 | 60 | Male | 69 | L | 2b |
| 0.013577982 | 289-9537671T | 1 | 12 | Male | 65 | U | 3c |
| 0.006960904 | 255-9532010T | 0 | 60 | Male | 64 | U | 2b |
| 0.234007837 | 515-9532963T | 1 | 38 | Male | 55 | U | 3b |
| 0.01146916 | 109-Z69-26351T | 1 | 36 | Male | 75 | L | 3b |
| 0.014579851 | 453-9541959T | 0 | 54 | Male | 66 | L | 2a |
| 0.014382125 | 303-9538803T | 1 | 5 | Femal | 50 | L | 2b |
| 0.011912985 | 269-9537501T | 0 | 9 | Male | 68 | M | 3b |
| 0.037668656 | 293-9537867T | 1 | 15 | Femal | 69 | M | 3c |
| 0.204786773 | 421-9541224T | 0 | 54 | Male | 51 | L | 2a |
| 0.007006292 | 93-Z135-29520T | 0 | 49 | Male | 69 | U | 3a |
| 0.06237981 | 317-9538998T | 0 | 60 | Femal | 68 | U | 2a |
| 0.029996877 | 21-A23-19954T | 0 | 60 | Male | 70 | M | 3c |
| 0.046211017 | 249-9536435T | 1 | 5 | Femal | 56 | L | 4 |
| 0.196504939 | 363-9539657T | 1 | 9 | Male | 81 | L | 4 |
| 0.13399581 | 231-9534192T | 1 | 17 | Male | 65 | U | 3c |
| 0.138340141 | 25-A09-18495T | 0 | 60 | Male | 60 | L | 2a |
| 0.033253613 | 327-9539112T | 0 | 47 | Male | 67 | U | 2a |
| 0.048954293 | 47-Z95-28404T | 1 | 36 | Male | 70 | M | 3c |
| 0.051538287 | 223-N23-21184T | 1 | 30 | Femal | 78 | L | 3b |

| | | | | | | | |
|---|---|---|---|---|---|---|---|
| 0.045647344 | 9-A03-15178T | 0 | 60 | Male | 68 | U | 2a |
| 0.046715334 | 341-9539270T | 0 | 25 | Male | 47 | L | 3c |
| 0.089303595 | 353-9539589T | 0 | 59 | Femal | 66 | L | 2a |
| 0.358431109 | 425-9541358T | 1 | 12 | Male | 73 | U | 3b |
| 0.002774187 | 295-9538577T | 0 | 60 | Male | 48 | L | 2b |
| 0.255841015 | 401-9540945T | 0 | 50 | Male | 69 | L | 3c |
| 0.131307226 | 125-A39-23912T | 0 | 8 | Male | 34 | U | 3b |
| 0.263630223 | 345-9532546T | 1 | 13 | Femal | 52 | L | 3c |
| 0.018190799 | 75-Z119-28790T | 0 | 48 | Femal | 54 | M | 3b |
| 0.062961529 | 493-9540853T | 1 | 12 | Male | 62 | U | 4 |
| 0.202941628 | 333-9532579T | 1 | 29 | Male | 63 | L | 3c |
| 0.259843623 | 69-N37-23830T | 1 | 8 | Male | 68 | U | 3c |
| 1.538732286 | 329-9532578T | 0 | 39 | Male | 52 | L | 3b |
| 0.01988476 | 507-9532687T | 1 | 22 | Male | 74 | U | 3b |
| 0.972127246 | 587-9547147T | 0 | 47 | Male | 62 | L | 3b |
| 9.7342107 | 589-9547138T | 1 | 12 | Femal | 41 | M | 4 |
| 0.17330784 | 355-9539603T | 1 | 30 | Male | 65 | U | 4 |
| 0.020892796 | 307-9538826T | 0 | 60 | Male | 38 | L | 2b |

**Supplementary Table 2. The probe sequence of RNA FISH**

| **Probe sequence name** | **Probe Sequence（5’-3’）** |
|---|---|
| Human TNFRSF10A-AS1_1 | GTTGCTATCACTGAATACCT |
| Human TNFRSF10A-AS1_2 | GGTTATTTGTATTAGTCTGT |
| Human TNFRSF10A-AS1_3 | AAAACACTTACGTGTGGCCG |
| Human TNFRSF10A-AS1_4 | AAGTACTTTTTTTTGGCGGG |
| Human TNFRSF10A-AS1_5 | ACCTTTGGAAATTTGGACTT |
| Human TNFRSF10A-AS1_6 | CCTTGAGTTCAAGTCACAAT |
| Human TNFRSF10A-AS1_7 | TGGCCGTCCAGTAAGCTAAG |
| Human TNFRSF10A-AS1_8 | AGTCAAGGGAACAGCATATA |

**Supplementary Table 3. The TNFRSF10A-AS1 sequence and the sgRNA position of CRISPR/Cas9 knockout assay**

| TNFRSF10A-AS1: |
| --- |
| AGGTATTCAGTGATAGCAACAGAAAACAGACTAATACAAATAACCATCAT<br>TTAACTTAGGTTAATATAAGATTTTTAAGTTACCAAAAATTAAATATAAAAA<br>TATTTAAAAGTAGACATCTTGGCTAGGTGCAGTGGCTCACGCCCATAATC<br>CCAGCACTTTGGGAGGCCGAGCCACCATGCCCGGCCACACGTAAGTGT<br>TTTGATTAGTAAATCCAGGTAGAAGTTGTATGTGTGAACTGTAAATAATGT<br>TGACAACTCTTAAGAATTGTCTGTTTTAATTAAACCAAGAAACTTAAACTA<br>GCTTTCTATTTACTAAAGATTATCTCAGATCACGTGACCTTGAAAAACATT<br>TAGATGGGCTCCAGTTTTTCTAAGAAAATGCTCCATTTATGGAAGCAATT<br>CTTTTCTTTCTTTTAACCAAATCTTTGCATAGGTACCAAATAACACATTTGT<br>TTAGGATGAGAGCTGCCCACTGCCCCCGCCAAAAAAAAGTACTTTTATAT<br>ACAAAAGTCCAAATTTCCAAAGGTATATGTACTTTAATTGTGACTTGAACT<br>CAAGGTAAATAAATTAAATAATTAATAAATTAACCTTAGCTTACTGGACGG<br>CCACCATCTTATATGCTGTTCCCTTGACTG |

| U6-sgRNA(TNFRSF10A-AS1)*4 |
| --- |
| GCTAGCGCTACCGGACTCAGGAGGGCCTATTTCCCATGATTCCTTCATA<br>TTTGCATATACGATACAAGGCTGTTAGAGAGATAATTAGAATTAATTTGAC<br>TGTAAACACAAAGATATTAGTACAAAATACGTGACGTAGAAAGTAATAATT<br>TCTTGGGTAGTTTGCAGTTTTAAAATTATGTTTTAAAATGGACTATCATAT<br>GCTTACCGTAACTTGAAAGTATTTCGATTTCTTGGCTTTATATATCTTGTG<br>GAAAGGACGAAACACCGGTAACAGTACTCAGACGTAGGTTTTAGAGCTA<br>GAAATAGCAAGTTAAAATAAGGCTAGTCCGTTATCAACTTGAAAAAGTGG<br>CACCGAGTCGGTGCTTTTTTACCGGTGAGGGCCTATTTCCCATGATTCCT<br>TCATATTTGCATATACGATACAAGGCTGTTAGAGAGATAATTAGAATTAAT<br>TTGACTGTAAACACAAAGATATTAGTACAAAATACGTGACGTAGAAAGTA<br>ATAATTTCTTGGGTAGTTTGCAGTTTTAAAATTATGTTTTAAAATGGACTAT<br>CATATGCTTACCGTAACTTGAAAGTATTTCGATTTCTTGGCTTTATATATC<br>TTGTGGAAAGGACGAAACACCGATTGCAATTTGTGCGTGATAGTTTTAGA<br>GCTAGAAATAGCAAGTTAAAATAAGGCTAGTCCGTTATCAACTTGAAAAA<br>GTGGCACCGAGTCGGTGCTTTTTTCTCGAGGAGGGCCTATTTCCCATGA<br>TTCCTTCATATTTGCATATACGATACAAGGCTGTTAGAGAGATAATTGGAA<br>TTAATTTGACTGTAAACACAAAGATATTAGTACAAAATACGTGACGTAGAA<br>AGTAATAATTTCTTGGGTAGTTTGCAGTTTTAAAATTATGTTTTAAAATGG<br>ACTATCATATGCTTACCGTAACTTGAAAGTATTTCGATTTCTTGGCTTTAT<br>ATATCTTGTGGAAAGGACGAAACACCGCTAAGAAAATGCTCCATTTAGTT<br>TTAGAGCTAGAAATAGCAAGTTAAAATAAGGCTAGTCCGTTATCAACTTG<br>AAAAAGTGGCACCGAGTCGGTGCTTTTTTGGATCCGAGGGCCTATTTCC<br>CATGATTCCTTCATATTTGCATATACGATACAAGGCTGTTAGAGAGATAAT<br>TGGAATTAATTTGACTGTAAACACAAAGATATTAGTACAAAATACGTGACG<br>TAGAAAGTAATAATTTCTTGGGTAGTTTGCAGTTTTAAAATTATGTTTTAAA |

ATGGACTATCATATGCTTACCGTAACTTGAAAGTATTTCGATTTCTTGGCT
TTATATATCTTGTGGAAAGGACGAAACACCGCTAATCAAAACACTTACGT
GGTTTTAGAGCTAGAAATAGCAAGTTAAAATAAGGCTAGTCCGTTATCAA
CTTGAAAAAGTGGCACCGAGTCGGTGCTTTTTTCTGCAGTCGACGGTAC
CGCGG

**Supplementary Table 4. DNA sequences of primers used in this study**

| Primer name | Sequence (5'-3') |
|---|---|
| β-actin-F | TGGACATCCGCAAAGACCTG |
| β-actin-R | CCGATCCACACGGAGTACTT |
| TNFRSF10A-AS1-F | TCTCAGATCACGTGACCTTGA |
| TNFRSF10A-AS1-R | GTGGGCAGCTCTCATCCTAA |
| MPZL1-F | GTTAAGCAGGCTCCTCGGAA |
| MPZL1-R | TCCGCATACACCACAGACTC |
| T-AS1-KO-F | CCAAGTTTGCAAGCACACGATTTC |
| T-AS1-KO-R | CTTCTCAACCTCCATTATCAGATGC |

**Supplementary Table 5. Antibodies used in this study**

| Antibodies | Source | Identifier |
|---|---|---|
| Anti-MPZL1 | Cell Signaling Technology | Cat#9893 |
| Anti-Cleaved Caspase-9 | Cell Signaling Technology | Cat#9509 |
| Anti-Cleaved Caspase-8 | Cell Signaling Technology | Cat#8592 |
| Anti-Cleaved Caspase-7 | Cell Signaling Technology | Cat#8438 |
| Anti-Caspase-9 | Cell Signaling Technology | Cat#9508 |
| Anti-Caspase-8 | Cell Signaling Technology | Cat#9746 |
| Anti-Caspase-7 | Cell Signaling Technology | Cat#9492 |
| Anti-Cyclin D1 | Cell Signaling Technology | Cat#2922 |
| Anti-CDK4 | Cell Signaling Technology | Cat#12790 |
| Anti-Snail | Cell Signaling Technology | Cat#3879 |
| Anti-Slug | Cell Signaling Technology | Cat#9585 |
| Anti-β-actin | Cell Signaling Technology | Cat#3700 |
| Ki-67 | Cell Signaling Technology | Cat#9449 |

**Supplementary Table 6 KEGG pathways enriched by differentially expressed genes affected by T-AS1.**

| pathway_ID | pathway_des | Diff gene | UP genes | down genes | Diff gene | all_gene in pathway | rich factor | Pvalue | Qvalue |
|---|---|---|---|---|---|---|---|---|---|
| hsa04668 | TNF signaling pathway | 6 | 2 | 4 | 145 | 130 | 2.8255 | 0.0137 | 0.3564 |
| hsa04115 | p53 signaling pathway | 4 | 1 | 3 | 145 | 74 | 3.3092 | 0.0170 | 0.3306 |
| hsa04151 | PI3K-Akt signaling pathway | 12 | 4 | 8 | 145 | 375 | 1.9590 | 0.0250 | 0.3076 |
| hsa04210 | Apoptosis | 6 | 2 | 4 | 145 | 153 | 2.4008 | 0.0310 | 0.3613 |
| hsa05226 | Gastric cancer | 6 | 2 | 4 | 145 | 162 | 2.2674 | 0.0407 | 0.4126 |
| hsa04630 | JAK-STAT signaling pathway | 6 | 1 | 5 | 145 | 167 | 2.1995 | 0.0469 | 0.4207 |
| hsa05202 | Transcriptional misregulation in cancer | 8 | 4 | 4 | 145 | 195 | 2.5116 | 0.0127 | 0.3725 |
